# Otogenic Sigmoid Sinus Thrombosis Complicated by Cerebellar Abscess and Hydrocephalus: A Case Report

**DOI:** 10.1002/ccr3.73589

**Published:** 2026-10-05

**Authors:** Alexandra Mădălina Bizdu‐Branovici, Luana Gherasie, Irina Gabriela Ionița, Maria Denisa Zica, Andreea Rusescu, Răzvan Hainaroșie, Viorel Zainea

**Affiliations:** ^1^ “Carol Davila” University of Medicine and Pharmacy Bucharest Romania; ^2^ “Prof. Dr. D. Hociotă” Institute of Phonoaudiology and Functional ENT Surgery Bucharest Romania

**Keywords:** brain abscess, hydrocephalus, mastoiditis, otogenic sigmoid sinus thrombosis, venous sinus thrombosis

## Abstract

A 44‐year‐old man developed otogenic sigmoid sinus thrombosis with cerebellar abscess, hydrocephalus, and recurrent posterior fossa infection. Staged neurosurgical drainage and canal‐wall‐down mastoidectomy achieved source control. Cultures were negative. Anticoagulant exposure was perioperative/early postoperative. Despite persistent venous non‐opacification, he was discharged improved, afebrile, without neurological deficit, and with remitted otoliquorrhea.

## Key Clinical Message

In adult otogenic sigmoid sinus thrombosis, neurological complications may precede otologic diagnosis. This case is distinctive for simultaneous cerebellar abscess, hydrocephalus, extensive venous thrombosis, visible intrasigmoid thrombus, culture‐negative purulence, perioperative anticoagulant use, and favorable discharge despite persistent venous non‐opacification.

## 1 Introduction

Otogenic sigmoid sinus thrombosis (otogenic SST) is an uncommon but severe intracranial complication of otomastoid infection. The older term “lateral sinus thrombosis” can be imprecise because the lateral sinus includes both the transverse sinus and the sigmoid sinus; therefore, the venous segments involved should be described anatomically whenever possible. Contemporary literature emphasizes that otogenic cerebral venous sinus thrombosis usually requires combined medical and surgical management, while the extent of direct sinus surgery and the role of anticoagulation remain debated [1, 2, 3, 4, 5, 6].

The pathogenic link between mastoid infection, sigmoid sinus thrombosis and posterior fossa complications is well recognized. The relevance of the present case lies not in a novel mechanism of spread, but in the complexity of management in an adult patient with cerebellar abscess, obstructive hydrocephalus, extensive sigmoid–transverse sinus and internal jugular thrombosis, culture‐negative microbiology, recurrent posterior fossa abscess, conservative sinus management despite visible intrasigmoid thrombus and favorable short‐term clinical discharge despite persistent venous non‐opacification [7, 8].

## 2 Case Report

A 44‐year‐old man with no documented major comorbidities was initially admitted to the neurosurgical service on 20 November 2024 with a left cerebellar abscess, obstructive hydrocephalus, and multiple intracranial venous sinus thromboses. Initial neuroimaging demonstrated the posterior fossa abscess and venous thrombotic involvement, prompting emergency external ventricular drainage and evacuation of the cerebellar abscess on the day of admission (Figure 1). Symptom duration and any preadmission outpatient antimicrobial exposure were not specified in the referral documentation.

**FIGURE 1:**
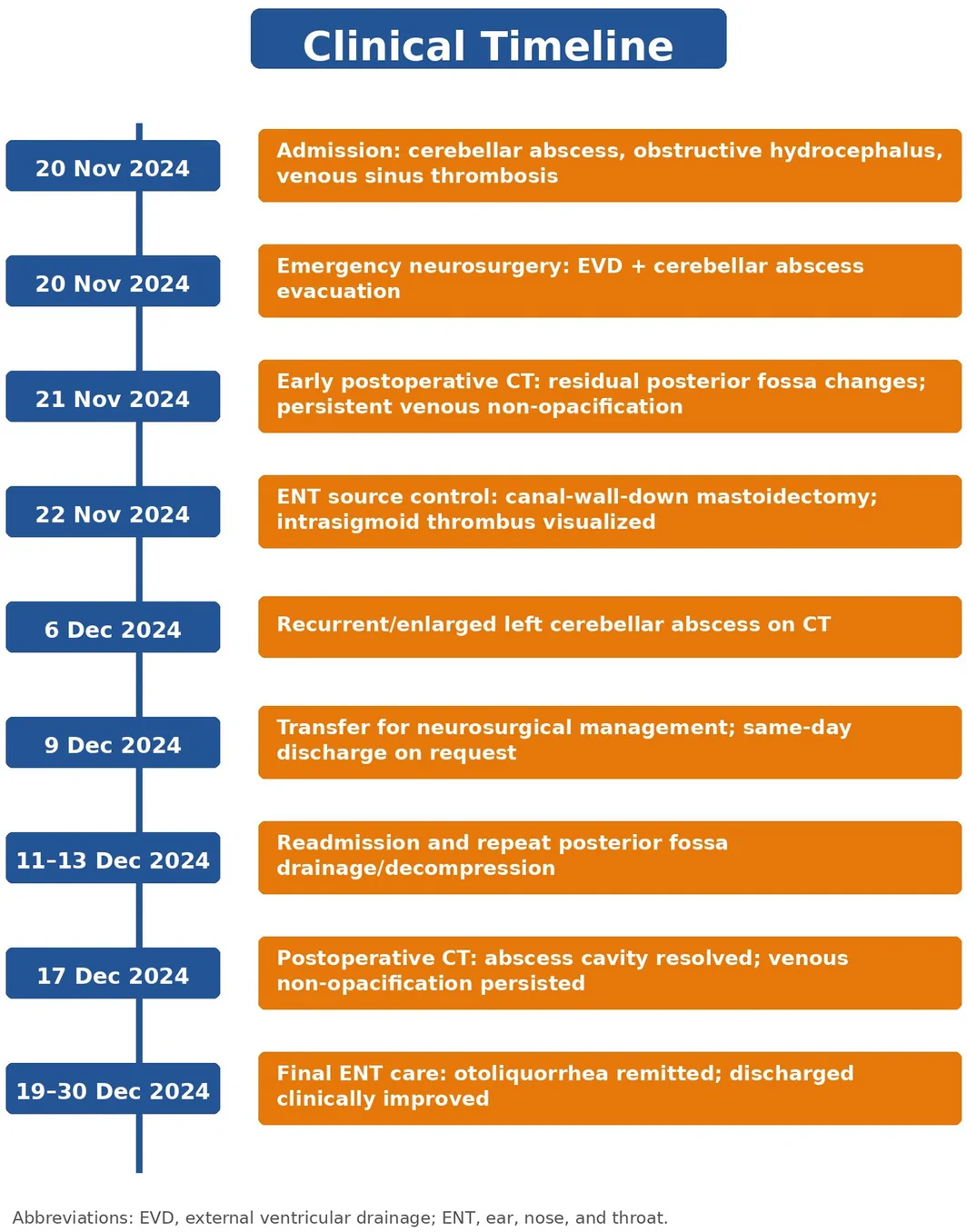
Clinical timeline of the patient's management. Chronological overview of the diagnostic and therapeutic interventions in a patient with otogenic sigmoid sinus thrombosis complicated by cerebellar abscess and hydrocephalus, including emergency neurosurgical drainage, subsequent radical mastoidectomy for eradication of the otogenic focus, recurrence of the cerebellar abscess, and second neurosurgical drainage, followed by recovery under antibiotic and anticoagulant therapy.

After the immediate neurosurgical emergency had been controlled, the patient was transferred intubated and mechanically ventilated to the ENT service specifically for surgical eradication of the suspected otogenic focus. The otogenic origin was supported by the association between posterior fossa infection, venous sinus thrombosis, documented left suppurative otomastoiditis and subsequent otologic findings (Figure 2). At otologic assessment, otomicroscopy showed a polypoid lesion in the left external auditory canal, mainly in the anteroinferior quadrant, with medial canal bulging; the visible portions of the tympanic membrane were described as intact. Formal audiometry was not feasible in the acute setting because the patient was intubated and mechanically ventilated and no quantitative audiogram from the preceding ENT service was available.

**FIGURE 2:**
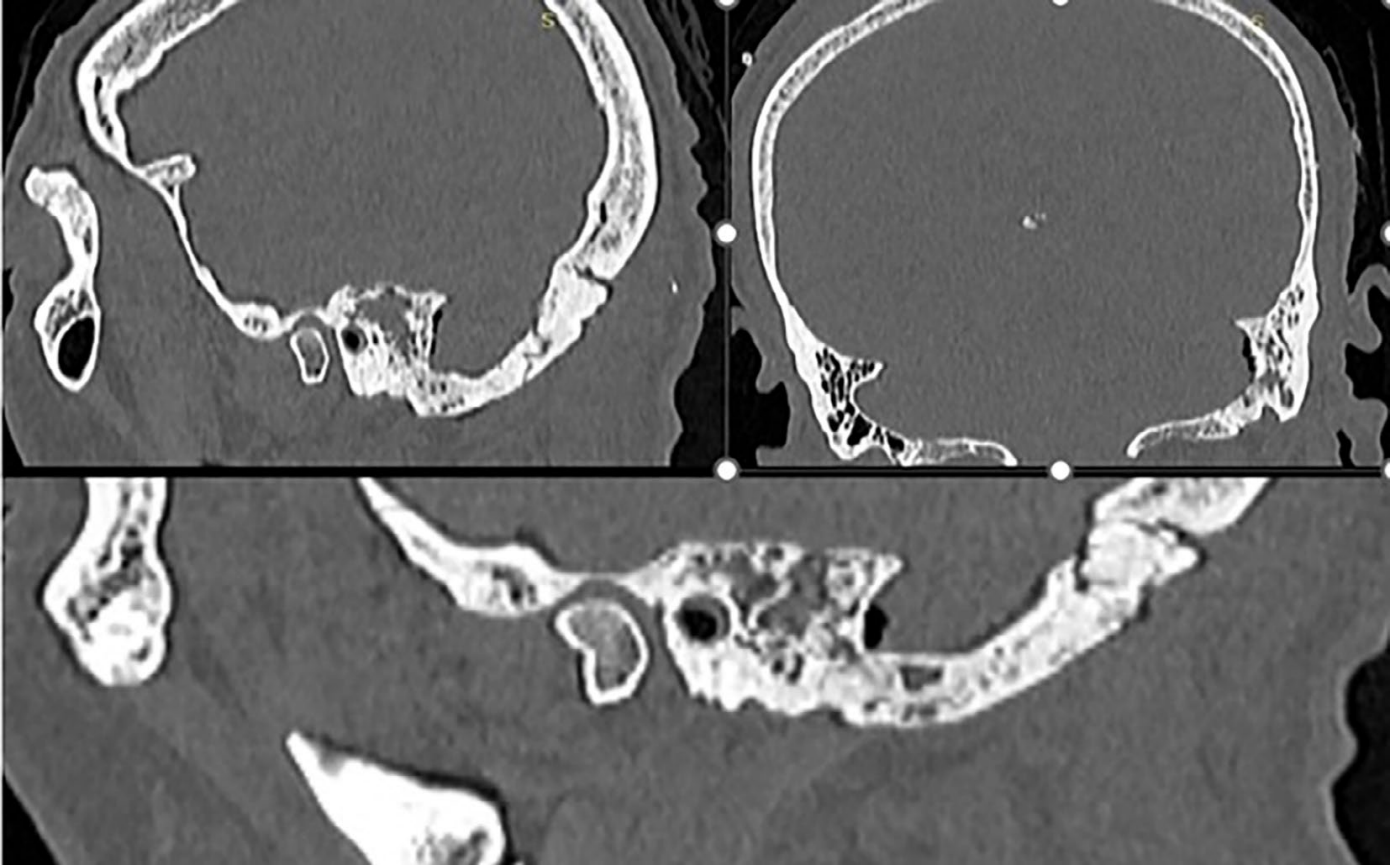
CT findings supporting left otomastoid disease as the suspected otogenic source. Sagittal and coronal high‐resolution CT of the temporal bone demonstrating opacification of the left mastoid air cells and middle ear cavity, consistent with otomastoiditis and suggesting an otogenic source of intracranial infection.

## 3 Methods

On postoperative day 1, contrast‐enhanced CT performed on 21 November 2024 showed residual left posterior fossa changes after occipital craniectomy, including a small residual cerebellar collection, perilesional edema with fourth‐ventricle compression, basal cistern effacement and mild inferior tonsillar descent. A right frontal ventricular drain was in place, with a small adjacent frontal intraparenchymal hematoma. The venous phase demonstrated persistent non‐opacification of the left internal jugular vein, left sigmoid sinus and left transverse sinus, with an additional partial filling defect at the distal right transverse sinus/confluence. In this report, venous involvement is described according to the available CT and contrast‐enhanced CT examinations. CT findings supporting left otomastoid involvement are shown in Figure 2.

On 22 November 2024, the patient underwent left canal‐wall‐down radical mastoidectomy for otologic source control. Intraoperatively, the mastoid cavity was occupied by chronically inflamed polypoid mucosa. The operative findings demonstrated destructive inflammatory disease, with partial ossicular‐chain erosion, dehiscence of the intratympanic facial nerve segment, and dehiscence of the sigmoid sinus plate. Through the dehiscent sigmoid plate, an intrasigmoid thrombus was directly visualized as shown in Figure 3. Purulent mastoid secretion was collected during mastoidectomy for bacteriological analysis, and tissue fragments were submitted for histopathological examination. Hemostasis was achieved with absorbable hemostatic material, and the mastoid cavity was packed with sterile endaural gauze.

**FIGURE 3:**
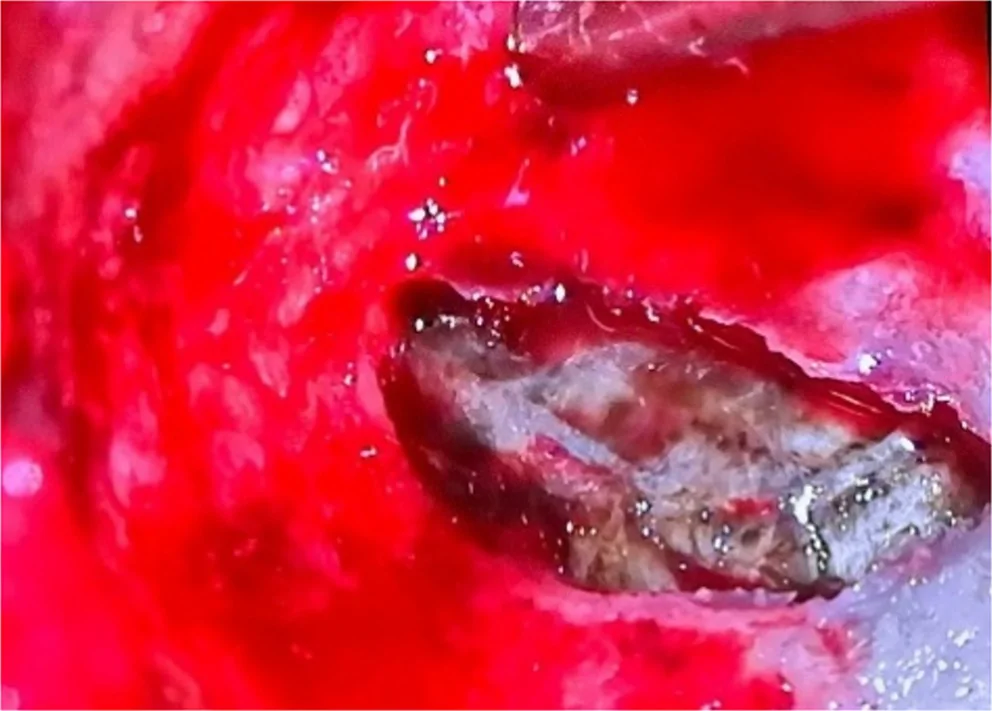
Intraoperative view demonstrating direct visualization of the sigmoid sinus thrombus through a dehiscent sigmoid plate.

The surgical objective at the sigmoid sinus was limited to exposure, assessment, and eradication of the adjacent otogenic septic focus. Although the thrombus was visible through the dehiscent sigmoid plate, the sinus was not incised, thrombectomy was not attempted, and internal jugular vein ligation was not performed. This conservative sinus strategy reflected the extent of venous thrombosis, the very recent neurosurgical intervention, and the perceived postoperative bleeding risk.

Histopathology of the otologic specimen showed stratified squamous epithelium with acanthosis and hyperkeratosis, associated with congestive chorion and chronic inflammatory infiltrate. Cholesteatoma was considered clinically because of the destructive otomastoid process, but it was not confirmed histopathologically. The otologic disease was therefore interpreted as severe suppurative polypoid otomastoiditis with destructive inflammatory changes rather than confirmed cholesteatomatous disease.

After mastoidectomy, the patient continued ENT‐directed postoperative care, including retroauricular wound care, mastoid cavity aspiration, local packing, and topical treatment. The early post‐mastoidectomy course was initially favorable, with reduction of otorrhea and vertigo. However, on 6 December 2024, follow‐up native and contrast‐enhanced cranial CT identified recurrence or enlargement of the left cerebellar abscess, measuring approximately 12 × 48 mm (Figure 4). Neurosurgical reassessment was requested, and transfer to neurosurgery was recommended.

**FIGURE 4:**
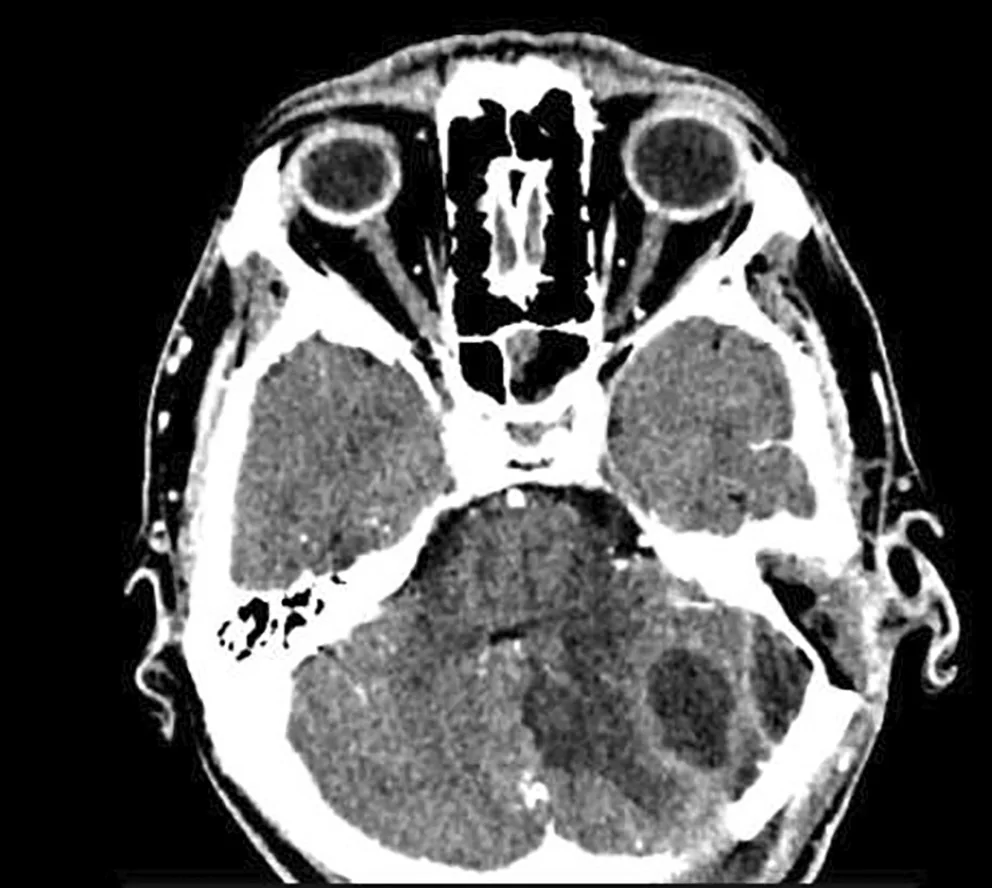
Cranial CT imaging revealed re‐expansion of a left cerebellar abscess, measuring approximately 12 × 48 mm associated with surrounding edema and mass effect, which prompted repeat neurosurgical drainage.

The patient was transferred for neurosurgical management on 9 December 2024 but requested same‐day discharge against medical advice. He was readmitted to the neurosurgical service on 11 December 2024 for recurrent left cerebellar abscess. During this second neurosurgical admission, repeat posterior fossa surgical management was performed; the hospital procedural record dated 13 December 2024 documented drainage of intracranial infection and posterior fossa decompression.

Microbiological results were reported according to the timing of sampling. Purulent secretion collected during mastoidectomy was tested for Staphylococcus spp., Streptococcus spp., Enterobacteriaceae, Pseudomonas spp., and Candida spp.; no Candida spp. and no pathogenic bacterial flora were identified after 48 h. Cerebrospinal fluid collected on 18 December 2024, after the second neurosurgical episode, was culture‐negative, with no organisms on Gram staining and no acid‐fast bacilli on Ziehl–Neelsen staining. During the final ENT admission, otic secretion and fungal cultures collected on 20 December 2024 were negative. Blood‐culture results and culture results from the initial cerebellar abscess drainage were not available in the reviewed records. Because no pathogen was isolated, antimicrobial susceptibility testing could not be performed.

The medication chart allowed reconstruction of the later inpatient antimicrobial exposure. After repeat drainage of the recurrent abscess, piperacillin–tazobactam 4.5 g every 8 h and amikacin 500 mg every 12 h were documented. Both agents were recorded for approximately 2 intensive‐care treatment days and 4 additional neurosurgical‐ward treatment days. During the subsequent ENT admission, piperacillin–tazobactam corresponded to approximately 12 additional treatment days, whereas amikacin corresponded to 3 additional treatment days. Overall, the reconstructible inpatient exposure comprised approximately 18 treatment days of piperacillin–tazobactam and approximately 9 treatment days of amikacin. Earlier Tazocin and amikacin administration was mentioned in discharge summaries from the first admission, but dose‐by‐dose reconstruction was possible only for the later inpatient course.

Available documentation showed perioperative and early postoperative anticoagulant exposure rather than a continuous standardized anticoagulation protocol. Unfractionated heparin was listed during the second neurosurgical intervention. In the postoperative intensive‐care setting, enoxaparin/Clexane 0.6 mL subcutaneously once daily was recommended and one 80 mg/0.8 mL syringe was recorded in the medication chart. Additional unfractionated heparin was later listed during the ENT admission, but the available records did not specify route, exact administered dose, frequency, indication, or total duration. No anti‐Xa monitoring, formal thrombophilia work‐up, or outpatient anticoagulation plan was available.

## 4 Conclusions and Results

### 4.1 Outcome and Follow Up

Postoperatively, the patient was monitored in intensive care from 13 to 15 December 2024. He was initially sedated, intubated, and mechanically ventilated. After sedation was reduced, he became conscious and cooperative, was successfully extubated, maintained spontaneous breathing with supplemental oxygen, remained hemodynamically stable, afebrile, and had preserved diuresis. He was subsequently transferred back to the neurosurgical ward.

Postoperative CT performed on 17 December 2024, compared with the 6 December examination, showed disappearance of the left cerebellar intraparenchymal abscess cavity, with reduction of edema and mass effect. However, persistent non‐opacification of the left internal jugular vein, left sigmoid sinus, and left transverse sinus was still present. A left subgaleal serous collection with possible wall enhancement and communication with the radical mastoidectomy cavity was also described and required monitoring. No spontaneous venous recanalization was documented on the available follow‐up imaging.

The patient was transferred back to the ENT service on 19 December 2024 for continued local postoperative care and remained hospitalized there until 30 December 2024. ENT examination documented serohematic secretion in the left external auditory canal and mastoid cavity, clinically interpreted as otoliquorrhea. During this final ENT admission, otoliquorrhea progressively diminished and was documented as remitted at discharge. The patient was discharged clinically improved, afebrile, without documented neurological deficit and with ear‐protection measures, head elevation, local wound‐care instructions, and a scheduled ENT outpatient reassessment. He did not attend the planned outpatient visit; therefore, no completed 3‐month clinical, audiological, or venographic follow‐up was available. Accordingly, outcome assessment is limited to short‐term inpatient clinical and radiological follow‐up.

## 5 Discussion

The present case illustrates the importance of staged source control and risk‐balanced decision‐making rather than a novel pathway of otogenic disease spread. Otogenic extension from mastoid infection to the sigmoid sinus and posterior fossa is a recognized mechanism. The educational value of this case lies in the adult presentation initially dominated by a neurosurgical emergency, the coexistence of multiple severe intracranial complications, culture‐negative purulent otomastoid disease, recurrent cerebellar abscess after initial source control, conservative sigmoid sinus management despite direct visualization of an intrasigmoid thrombus and perioperative/early postoperative anticoagulant exposure in a patient with relevant bleeding risk [1, 2, 3, 4].

The chronology supports the staged therapeutic sequence used in this patient. Emergency posterior fossa drainage and cerebrospinal fluid diversion were prioritized because obstructive hydrocephalus and posterior fossa mass effect represented immediate life‐threatening conditions. After neurosurgical stabilization, the patient was transferred intubated and mechanically ventilated to the ENT service for surgical eradication of the suspected otogenic focus. Canal‐wall‐down mastoidectomy was appropriate in the context of destructive suppurative otomastoiditis, sigmoid plate dehiscence and direct intraoperative visualization of an intrasigmoid thrombus. Contemporary literature on otogenic cerebral sinus thrombosis supports combined medical and surgical management, although the extent of direct sinus surgery and the role of anticoagulation remain debated [1, 2, 3, 4].

The conservative approach to the sigmoid sinus is clinically defensible in this setting. Historically, management of otogenic sigmoid sinus thrombosis often included sinus incision, thrombectomy, or internal jugular vein ligation. Contemporary reports tend to reserve direct sinus procedures for selected cases, such as persistent septicemia, septic embolization, progressive thrombosis despite treatment, or failure of source control [4, 5, 6, 7, 8]. In the present case, the sigmoid sinus was exposed and the thrombus was directly visualized through the dehiscent sigmoid plate, but the sinus was not incised, thrombectomy was not attempted, and internal jugular vein ligation was not performed. Given the extensive venous thrombosis, very recent posterior fossa surgery, postoperative hematoma risk, and absence of documented persistent septicemia at that stage, otologic source control without direct sinus intervention represented a reasonable risk‐balanced strategy.

The culture‐negative microbiological profile is central to the interpretation of antimicrobial therapy. Brain abscess guidelines and recent infectious‐disease practice data emphasize pathogen identification, susceptibility testing, and follow‐up imaging as important components of management [9, 10]. In this case, however, no positive isolate was documented from the available microbiological records; therefore, antimicrobial susceptibility testing could not be performed, and treatment could not be narrowed microbiologically. Piperacillin–tazobactam combined with amikacin provided broad empirical coverage for severe otogenic infection, including Gram‐negative organisms and Pseudomonas risk. The recurrence of the cerebellar abscess cannot be attributed with certainty to a single factor. Plausible contributors include advanced disease at presentation, residual infected collection, impaired venous drainage, culture‐negative microbiology limiting targeted therapy, and the intrinsic difficulty of achieving source control across both otologic and posterior fossa compartments.

Anticoagulation in otogenic sigmoid sinus thrombosis remains controversial. Systematic and narrative reviews emphasize that the available evidence is largely observational and that randomized trials are lacking [1, 2, 3]. Potential benefits include prevention of thrombus propagation and facilitation of venous recanalization, whereas the risks are particularly relevant after neurosurgical intervention or in the presence of postoperative hemorrhagic complications [5, 6, 7, 8]. The present case should therefore not be interpreted as supporting routine anticoagulation. The available records documented perioperative and early postoperative anticoagulant exposure, but not a continuous, fully reconstructible, monitored therapeutic anticoagulation protocol. Follow‐up CT did not demonstrate venous recanalization; therefore, recanalization should be discussed as a potential benefit reported in the literature rather than as an outcome observed in this patient.

The radiological follow‐up illustrates an important distinction between clinical recovery and venous outcome. Postoperative CT showed resolution of the left cerebellar abscess cavity, reduction of edema and decreased mass effect, supporting favorable short‐term intracranial evolution after repeat posterior fossa drainage. However, persistent non‐opacification of the left internal jugular vein, sigmoid sinus and transverse sinus remained present. MRI/MRV is generally preferred for direct thrombus characterization and venous follow‐up, and recent literature on mastoiditis‐associated cerebral sino‐venous thrombosis also highlights the importance of timely imaging and follow‐up imaging, particularly when anticoagulation decisions and duration are considered [11]. In this case, venous assessment was based on the available CT and contrast‐enhanced CT examinations; consequently, the manuscript reports persistent venous non‐opacification and does not infer delayed recanalization.

Short‐term clinical outcome was favorable despite the absence of documented venous recanalization. At discharge, otoliquorrhea had remitted, the patient was afebrile, and no neurological deficit was documented. A scheduled outpatient reassessment was planned, but the patient did not attend; consequently, no completed 3‐month clinical, audiological, or venographic follow‐up was available. Such reassessment would have been valuable to document neurological stability, hearing outcome, middle‐ear status, and possible delayed venous recanalization. The available outcome should therefore be interpreted as favorable short‐term inpatient clinical and radiological evolution rather than as documented long‐term recovery.

The interpretation of this case is limited by the retrospective nature of the available documentation and by the absence of long‐term follow‐up. The reviewed documentation did not allow complete reconstruction of the prehospital course or outpatient antibiotic exposure before admission. Blood‐culture results and culture results from the initial cerebellar abscess drainage were not available. Microbiological cultures available for review remained negative, preventing susceptibility‐guided antimicrobial therapy. Dose‐by‐dose antimicrobial reconstruction was possible only for the later inpatient course and heparin entries were incompletely specified in terms of route, exact administered dose, indication, frequency and total duration. No formal thrombophilia work‐up, quantitative audiogram, MRI/MRV report, separate infectious‐disease consultation note, complete outpatient anticoagulation plan or completed 3‐month follow‐up was available. These limitations reduce the strength of conclusions regarding optimal antimicrobial duration, anticoagulation strategy, venous recanalization and long‐term functional outcome, but they do not diminish the clinical relevance of the staged multidisciplinary management and favorable short‐term discharge status.

Overall, this case reinforces that otogenic sigmoid sinus thrombosis may present primarily as a neurological emergency, with posterior fossa abscess, obstructive hydrocephalus, and extensive venous thrombosis preceding or overshadowing the otologic diagnosis. In line with contemporary literature, management required staged neurosurgical and otologic source control, broad empirical antimicrobial therapy in a culture‐negative setting, conservative sigmoid sinus management, and cautious perioperative/early postoperative anticoagulant exposure in the context of recent neurosurgery and bleeding risk [1, 2, 3, 4]. Although short‐term CT did not demonstrate venous recanalization, the patient had a favorable clinical status at discharge, with remission of otoliquorrhea, absence of fever, and no documented neurological deficit. The case adds practical value by illustrating that favorable early clinical evolution may occur even when microbiology remains culture‐negative and venous recanalization is not yet demonstrated, provided that urgent source control, careful imaging surveillance, transparent reporting of treatment exposure, and individualized risk‐balanced anticoagulant decisions are prioritized [1, 2, 9, 10, 11, 12, 13, 14, 15, 16].

## Author Contributions

**Alexandra Mădălina Bizdu‐Branovici:** conceptualization, data curation, writing – original draft. **Luana Gherasie:** data curation. **Irina Gabriela Ionița:** writing – review and editing. **Maria Denisa Zica:** data curation, writing – review and editing. **Andreea Rusescu:** writing – review and editing. **Răzvan Hainaroșie:** data curation, writing – review and editing, supervision. **Viorel Zainea:** supervision, conceptualization, writing – review and editing.

## Funding

The authors have nothing to report.

## Ethics Statement

Written informed consent was obtained from the patient for publication of this case report and accompanying images. Ethical approval was not required for this study, in accordance with institutional policies for anonymized case reports.

## Conflicts of Interest

The authors declare no conflicts of interest.

## Data Availability

The data that support the findings of this study are available on request from the corresponding author. The data are not publicly available due to privacy or ethical restrictions.

## References

[1] M. George , A. A. Kolethekkat , E. Torrigiani , S. S. Marston , C. M. Thomas , and M. Raphael , “Management of Otogenic Sigmoid Sinus Thrombosis: A Systematic Review on the Role of Anticoagulation and Its Outcome,” Indian Journal of Otolaryngology and Head & Neck Surgery 75, no. 2 (2023): 450–456, 10.1007/s12070-022-03289-6. PMC10235279 37275038

[2] C. Picton , J. Rassam , C. Lloyd‐Davies , M. Gajre , and I. McKay‐Davies , “An Update on the Management of Otogenic Cerebral Sinus Thrombosis: A Systematic Review,” Clinical Otolaryngology 51, no. 4 (2026): 494–503, 10.1111/coa.70107. 41960854

[3] M. Kotowski and J. Szydlowski , “Otogenic Cerebral Sinus Thrombosis in Children: A Narrative Review,” Neurology and Therapy 12, no. 4 (2023): 1069–1079, 10.1007/s40120-023-00499-0. PMC10310653 37266793

[4] K. Raja , P. K. Parida , A. Alexander , and G. Surianarayanan , “Otogenic Lateral Sinus Thrombosis: A Review of Fifteen Patients and Changing Trends in the Management,” International Archives of Otorhinolaryngology 22, no. 3 (2018): 208–213, 10.1055/s-0037-1,604,198. PMC6033598 29983756

[5] M. Mather , K. Musgrave , and N. Dawe , “Is Anticoagulation Beneficial in Acute Mastoiditis Complicated by Sigmoid Sinus Thrombosis?,” Laryngoscope 128, no. 11 (2018): 2435–2436, 10.1002/lary.27151. 29521448

[6] M. L. Castellazzi , G. M. Di Pietro , M. Gaffuri , et al., “Pediatric Otogenic Cerebral Venous Sinus Thrombosis: A Case Report and a Literature Review,” Italian Journal of Pediatrics 46, no. 1 (2020): 122, 10.1186/s13052-020-00882-9. PMC7470606 32883359

[7] S. C. Prasad , S. H. Shin , A. Russo , G. Di Trapani , and M. Sanna , “Current Trends in the Management of the Complications of Chronic Otitis Media With Cholesteatoma,” Current Opinion in Otolaryngology & Head and Neck Surgery 21, no. 5 (2013): 446–454, 10.1097/MOO.0b013e3283646467. 23892792

[8] N. O. Penido , A. Borin , L. C. N. Iha , et al., “Intracranial Complications of Otitis Media: 15 Years of Experience in 33 Patients,” Otolaryngology and Head and Neck Surgery 132, no. 1 (2005): 37–42, 10.1016/j.otohns.2004.08.007. 15632907

[9] J. Bodilsen , Q. G. D'Alessandris , H. Humphreys , et al., “European Society of Clinical Microbiology and Infectious Diseases Guidelines on Diagnosis and Treatment of Brain Abscess in Children and Adults,” Clinical Microbiology and Infection 30, no. 1 (2024): 66–89, 10.1016/j.cmi.2023.08.016. 37648062

[10] R. Habis , M. Canales , S. E. Beekmann , et al., “Perspectives in Brain Abscess Diagnosis and Management: A National Survey of Infectious Disease Specialists,” Open Forum Infectious Diseases 12, no. 8 (2025): ofaf358, 10.1093/ofid/ofaf358. PMC12341925 40799790

[11] Y. Sellam , E. Zloczower , M. Gete , et al., “Acute Mastoiditis‐Associated Cerebral Sinus Venous Thrombosis in Children: A Retrospective Review,” European Journal of Pediatrics 184 (2025): 654, 10.1007/s00431-025-06477-y. PMC12491111 41039147

[12] K. B. Memis and S. Aydin , “Role of Imaging in Chronic Otitis Media and Its Complications,” World Journal of Radiology 17, no. 8 (2025): 109447, 10.4329/wjr.v17.i8.109447. PMC12400283 40901344

[13] U. Osma , S. Cureoglu , and S. Hosoglu , “The Complications of Chronic Otitis Media: Report of 93 Cases,” Journal of Laryngology and Otology 114, no. 2 (2000): 97–100, 10.1258/0022215001905012. 10748823

[14] L. H. Govea‐Camacho , R. Pérez‐Ramírez , A. Cornejo‐Suárez , R. Fierro‐Rizo , C. J. Jiménez‐Sala , and C. S. Rosales‐Orozco , “Diagnosis and Treatment of the Complications of Otitis Media in Adults. Case Series and Literature Review,” Cirugia y Cirujanos (English Edition) 84, no. 5 (2016): 398–404, 10.1016/j.circen.2016.08.001. 26738650

[15] Z. B. Khuzwayo and B. Enicker , “Otogenic Intracranial Complications: A 10‐Year Retrospective Review in KwaZulu‐Natal, South Africa,” Journal of Laryngology and Otology 134, no. 1 (2020): 3–7, 10.1017/S0022215120000018. 31964445

[16] P. Verma , A. Gargava , S. Saxena , and V. P. Narvey , “To Study the Prevelance of Extracranial & Intracranial Complications in Chronic Suppurative Otitis Media,” Indian Journal of Otolaryngology and Head & Neck Surgery 74, no. Suppl 1 (2022): 608–611, 10.1007/s12070-021-02423-0. PMC9411429 36032862

